# Haematuria: An Imaging Guide

**DOI:** 10.1155/2014/414125

**Published:** 2014-07-17

**Authors:** Fiachra Moloney, Kevin P. Murphy, Maria Twomey, Owen J. O'Connor, Michael M. Maher

**Affiliations:** Department of Radiology, Cork University Hospital, Wilton, Cork, Ireland

## Abstract

This paper discusses the current status of imaging in the investigation of patients with haematuria. The physician must rationalize imaging so that serious causes such as malignancy are promptly diagnosed while at the same time not exposing patients to unnecessary investigations. There is currently no universal agreement about the optimal imaging work up of haematuria. The choice of modality to image the urinary tract will depend on individual patient factors such as age, the presence of risk factors for malignancy, renal function, a history of calculus disease and pregnancy, and other factors, such as local policy and practice, cost effectiveness and availability of resources. The role of all modalities, including conventional radiography, intravenous urography/excretory urography, ultrasonography, retrograde pyelography, multidetector computed tomography urography (MDCTU), and magnetic resonance urography, is discussed. This paper highlights the pivotal role of MDCTU in the imaging of the patient with haematuria and discusses issues specific to this modality including protocol design, imaging of the urothelium, and radiation dose. Examination protocols should be tailored to the patient while all the while optimizing radiation dose.

## 1. Introduction

Haematuria can be microscopic or macroscopic (visible to the naked eye) in nature, but both forms may be the sole manifestation of underlying serious pathology. Haematuria is most accurately defined as the presence of three or more red blood cells per high-powered field in two of three properly collected urinalysis specimens [1, 2]. It may be symptomatic or asymptomatic and occur in isolation or in association with other urinary tract abnormalities [3].

The more common causes of haematuria include urinary tract infection, urolithiasis, trauma, renal parenchymal disease, and malignancy [4]. The most common primary malignancies associated with haematuria are renal cell carcinoma, urothelial cell carcinoma, prostate carcinoma, and, less commonly, squamous cell carcinoma [5]. Renal cell carcinoma accounts for 92% of all renal neoplasms with urothelial carcinoma representing 7% of upper urinary tract malignancies [6]. The majority of urothelial lesions occur in the bladder but synchronous lesions occur in 2% of renal urothelial lesions and 9% of ureteric lesions [7]. Due to the multifocal nature of urothelial carcinoma, an evaluation of the urothelium, including thorough evaluation of the upper urinary tracts, is warranted and represents a significant diagnostic challenge to the clinician. This paper discusses the current status of imaging in patients suspected of having a urological cause of haematuria. The evaluation of patients presenting with haematuria in the context of trauma, glomerular disease, and infection is beyond the scope of this review.

The role of all modalities, including conventional radiography, intravenous urography (IVU)/excretory urography, ultrasonography, retrograde pyelography, multidetector computed tomography urography (MDCTU), and magnetic resonance urography (MRU), is discussed. This paper highlights the pivotal role of MDCTU in the imaging of the patient with haematuria and discusses issues specific to this modality including protocol design, imaging of the urothelium, and radiation dose.

## 2. Investigation of Haematuria

The initial decision is to determine if all patients presenting with haematuria require imaging evaluation. The evaluation of haematuria should begin with a search for potential benign causes including menstruation, vigorous exercise, sexual activity, and infection [1]. In the absence of infection, the next step is to distinguish glomerular and nonglomerular causes of haematuria. The presence of proteinuria, red cell casts, dysmorphic red blood cells, or an elevated creatinine suggests a glomerular cause [1]. If the findings suggest a glomerular source of bleeding, urological referral is not required initially and referral to a nephrologist for further management is warranted [1, 8]. If a glomerular source is excluded, urological referral is indicated. The choice to investigate asymptomatic microscopic haematuria remains controversial. The prevalence of asymptomatic microscopic haematuria in the general population has been reported as varying between 0.2% and 21% [9]. Patients without a discernable urinary tract abnormality may normally release small amounts of red blood cells into urine so that one or two red blood cells may be visible at microscopy. Furthermore, microscopic haematuria can be intermittent, even in patients in which a malignancy is subsequently detected [10]. The low prevalence of significant urological malignancy in young patients with asymptomatic microscopic haematuria has led many authors to suggest that no imaging evaluation is necessary in this subgroup [11].

Macroscopic haematuria conveys a much higher risk of malignancy and warrants prompt investigation in all cases [12]. Urinary tract malignancy is four times more common in patients with macroscopic haematuria than microscopic haematuria with gross haematuria being the presenting symptom in 80% of bladder cancers and half of all renal cancers [13]. The prevalence of malignancy was 10% and 34.5% amongst patients under and over 50 years of age, respectively, in one series [14]. Therefore patients with macroscopic haematuria require complete evaluation of the upper and lower urinary tracts with upper urinary tract imaging and cystoscopy to exclude neoplasia.

Risk factors for urinary tract malignancy include smoking, occupational exposure to benzenes or aromatic amines, recurrent urinary tract infections, a history of irritative voiding symptoms, cyclophosphamide intake, pelvic irradiation, and analgesic abuse [1].

According to the British Association of Urological Surgeons, patients with macroscopic haematuria, symptomatic microscopic haematuria, and those over 40 years of age or with other risk factors with asymptomatic microscopic haematuria should be referred for urological investigation [15].

Haematuria in patients receiving oral antiplatelet agents or anticoagulants is often attributed to excessive anticoagulation. However, an underlying malignancy was found in 24% and 7% of patients in two separate series and thorough urological evaluation should not be foregone in patients receiving anticoagulants [16, 17].

The physician must rationalize the investigation of haematuria so that serious causes such as malignancy are promptly diagnosed while at the same time not exposing patients to unnecessary investigations with the potential for adverse events and unwanted consequences such as anxiety and radiation dose.

Complete urological evaluation for haematuria includes a full history and physical examination, laboratory analysis, and radiological imaging of the upper urinary tract followed by cystoscopic examination of the urinary bladder [1]. Urinary cytology, although controversial, often constitutes part of the initial work up for hematuria [18]. However, it has a high false negative rate for the detection of malignancy with a reported sensitivity of only 25% [19].

Furthermore, as negative cytology can never completely exclude the presence of a bladder tumor, cystoscopy is warranted in all cases [1]. Cytology may be of use in the identification of patients that warrant intense investigation.

## 3. Radiological Evaluation of Hematuria

The primary role of imaging is to identify those patients with a malignant cause of haematuria. Currently no imaging modality is sufficiently sensitive for the detection of urothelial carcinoma of the bladder and cystoscopic evaluation of the lower urinary tract is essential in the thorough evaluation of haematuria [14]. The choice of modality to image the upper urinary tract will depend on individual patient factors such as age, the presence of risk factors for malignancy, renal function, a history of calculus disease and pregnancy, and other factors, such as local policy and practice, cost effectiveness, and availability of resources.

There is no universal agreement about the optimal imaging work up of haematuria. Several protocols for the investigation of haematuria have been developed but they vary considerably and a lack of robust data from randomized control trials makes it difficult to formulate evidence-based guidelines [1, 12].

First-line investigations often include conventional radiography, renal ultrasound, and/or IVU in combination with cystoscopy. Second-line investigations include MDCTU and MRU, often only carried out if the first-line tests reveal an abnormality [14].

Previous protocols that incorporated a combination of renal ultrasound and IVU are now being superseded by MDCTU, which represents a one-stop diagnostic technique for the patient [20]. MDCTU is the most sensitive and specific test for the diagnosis of urinary tract calculi and for detecting and characterizing renal masses [21]. MDCTU has been shown to be more sensitive, specific, and accurate than IVU in the diagnosis of upper urinary tract urothelial carcinoma in patients with haematuria [22]. Furthermore, MDCTU has the advantage of imaging the periureteric tissues and retroperitoneum while excretory urography only images the ureteric lumen and cannot adequately depict any extrinsic abnormalities [23]. The superior diagnostic performance of MDCTU over IVU in the detection of malignancy, combined with recent advances in dose reduction and a wider availability of the technique, has led many authors to recommend MDCTU as a first-line modality to image the upper urinary tract in patients with haematuria.

The role of MRU is also increasing, although it is currently inferior to MDCTU in the detection of urothelial malignancy [24].

## 4. Conventional Radiography

The kidneys, ureters, and bladder (KUB) radiograph is currently of little value in the investigation of patients with haematuria [25].

Traditionally, the main role of radiography has been in the detection of calculus disease despite only moderate sensitivities in the range of 45 to 60% [26]. KUB cannot visualize radiolucent calculi (10–20% of calculi) and overlying bowel gas, faecoliths, and phleboliths can make interpretation difficult [25].

CT KUB or “stone protocol CT” is now the first-line imaging investigation in the diagnosis of calculus disease with a reported sensitivity of 96% to 100% and a specificity of 94% to 100% [27, 28]. The major limitation to the initial universal acceptance of CT KUB as a first-line test was the significantly higher radiation dose incurred by the patient. However, low-dose and ultralow-dose CT protocols have reduced radiation exposure by approximately 50% and 95%, respectively, compared with standard-dose CT, with comparable detection rates of calculi [29, 30]. Kluner et al. reported ultralow-dose CT with a high diagnostic accuracy obtained at doses equivalent to that of a conventional radiograph (0.5 mSv in men and 0.7 mSv in women) [30]. Furthermore, CT can assess for the presence of hydronephrosis or identify alternative causes of the patient's haematuria and extraurinary pathology. The reported incidence of extraurinary pathology with CT performed for suspected calculus disease is 12% [31].

KUB has no role in the detection of renal and urothelial carcinoma due to its limited sensitivity. Evidence of a malignancy such as calcification within a urothelial carcinoma may occasionally be provided [32]. Approximately 15% of renal cell carcinomas contain calcifications that are visible on conventional radiographs [33]. In general, radiography should be avoided, especially in high-risk patients, as further imaging will almost always be required [2].

## 5. Intravenous Urography

Since it was first performed by Osborne et al. in 1923 [34], intravenous urography (IVU) has been the traditional “gold standard” in the evaluation of the upper urinary tract [35]. Its main strength is that it images the entire upper urinary tract with a high degree of spatial resolution allowing detection of even small urothelial tumors. It has a reported sensitivity of 60.5% and specificity of 90.9% for the detection of urological abnormalities in patients with haematuria [36]. It also provides structural information as well as limited functional data, is widely available, and is often the most cost-efficient test in many centers. Its disadvantages include a lengthy acquisition time, the radiation dose involved, and a risk of contrast reaction [3]. Mean effective radiation doses in the range of 3.6 mSV to 9.6 mSv have been reported with IVU [37, 38]. In contrast, a three-phase CTU has a mean effective dose of 14.8 mSv plus or minus 9 mSv, corresponding to a dose 1.5 times higher than IVU [38].

**Figure 1:**
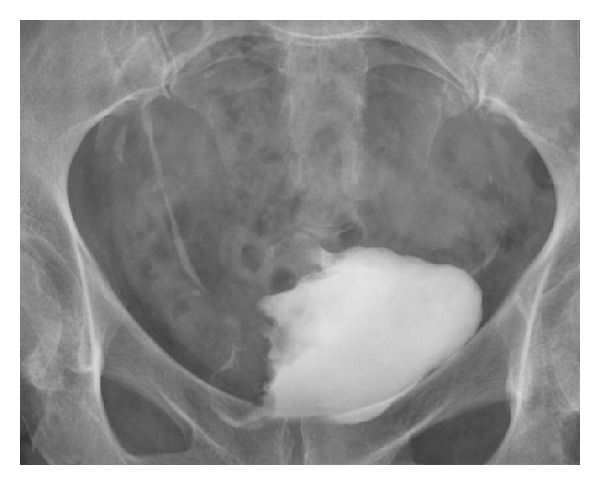
Intravenous urogram (bladder image obtained 15 minutes following contrast administration). There is an infiltrative mass lesion involving the bladder wall on the right. This was confirmed to be a urothelial cell carcinoma following biopsy at cystoscopy.

**Figure 2:**
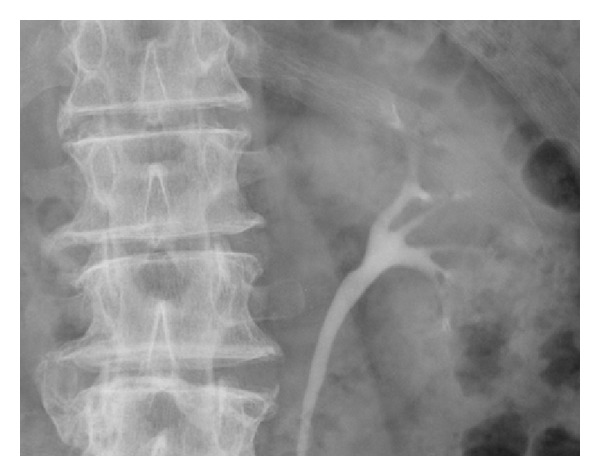
Intravenous urogram demonstrates a filling defect in the lower pole calyx of the left kidney, a histologically-proven urothelial cell carcinoma.

IVU can also depict renal masses although it is not possible to distinguish a cyst from a solid mass and many lesions can go undetected (Figures 1 and 2) [39]. It has a limited sensitivity for detecting renal masses less than 3 cm in size (reported sensitivities of 21% for masses less than 2 cm and 52% for masses 2 to 3 cm in size) [40] and, even when a mass is identified, further imaging, usually with CT, is required to characterize the lesion.

Current evidence casts doubt on the role of IVU as the “gold standard” for urothelial imaging and in recent years the dominant role of IVU has been surpassed by MDCTU [41]. MDCTU has been shown to be more accurate in the diagnosis of upper urinary tract urothelial carcinoma with a sensitivity of 96% and a specificity of 100% compared to IVU with a sensitivity of 75% and a specificity of 86% [22]. A filling defect in the renal pelvis or ureter on an IVU may be due to a neoplasm, calculus, blood clot, or possibly a vascular impression, thus limiting accurate interpretation without additional imaging [42]. CT has the advantage of imaging the urinary tract and retroperitoneum concurrently, thus leading to a higher diagnostic accuracy.

In the investigation of suspected urolithiasis, CT KUB is now a well-accepted alternative to IVU with superior calculus detection rates reported in several studies [43]. A potential limitation of CT is its inability to provide functional data that would otherwise be derived from the excretory times during an IVU. However, some authors have suggested that secondary measures of obstruction such as hydroureter, hydronephrosis, and perinephric fat stranding are a reliable indicator of the degree of obstruction within a system and comparable to delayed excretion on an IVU [44].

As with any imaging modality, the protocol should ideally be tailored to answer a specific clinical question. A standard IVU protocol includes a preliminary KUB radiograph prior to intravenous contrast administration which is followed by a nephrotomogram or nephrographic images collimated to the kidneys (1 to 3 minutes after contrast injection), a KUB radiograph at 5 minutes after injection, a pyelographic image at 10 minutes with abdominal compression applied after the 5-minute film and ureter-bladder images after compression is released, and bladder images. Additional films such as oblique images and additional tomograms may be obtained at the discretion of the reporting radiologist [42].

The preliminary KUB is to identify renal and ureteric calculi that may not be visible following contrast administration.

Multiple patterns of abnormality on IVU have been described with urethelial carcinoma. These include smooth or irregular ureteric filling defects seen in 35% of cases, filling defects within dilated calices secondary to partial or complete obstruction in 26%, or caliceal amputation in 19% of cases.

Mass lesions typically present with increased parenchymal thickness with associated underlying calyceal distortion [42].

## 6. Ultrasound

The primary advantage of ultrasound is the avoidance of exposure to ionizing radiation. Thus, it is especially useful in radiation-sensitive populations such as pregnant women and pediatric patients. It is also a widely available and inexpensive imaging modality and does not involve the administration of intravenous contrast.

The sensitivity of ultrasound is variable depending on the skill and experience of the operator and on the body habitus of the patient.

**Figure 3:**
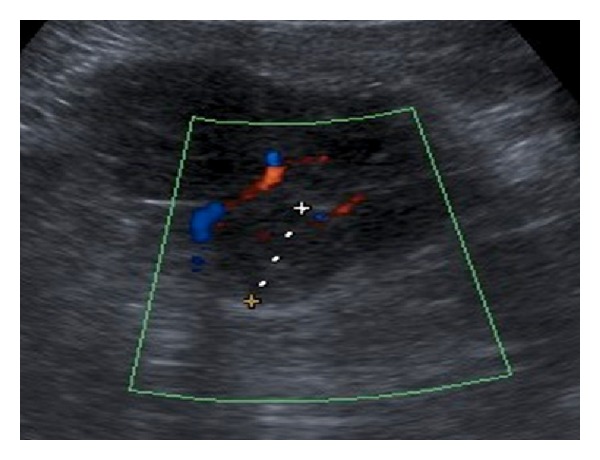
Renal ultrasound demonstrates an exophytic hypoechoic solid mass arising from the lower pole of the kidney consistent with a renal cell carcinoma.

Ultrasound is superior to IVU for the detection of renal masses with reported sensitivities of 67% and 79% for IVU and ultrasound, respectively [45, 46]. However, ultrasound has a limited sensitivity for the detection of small renal masses and is inferior to CT (Figure 3). Jamis-Dow et al. reported that for masses <1 cm, ultrasound detected 20% and CT detected 76% and, for masses <2 cm, ultrasound detected 70% and CT detected 95%. For masses >3 cm, the results were comparable [47]. Ultrasound may be more useful for lesion characterization rather than detection as it can accurately distinguish cystic from solid masses [11]. It is excellent for defining the internal architecture of a renal mass and determining the Bosniak grade, which guides management and prognosis [48]. Ultrasound has been reported to be the most cost effective method for the assessment of renal masses detected at IVU as over 80% of these masses are found to be simple cysts [49].

The major disadvantage of ultrasound is its limited ability to thoroughly evaluate the urothelium for malignancy. Detection of renal pelvis carcinoma is moderate (82%) but sensitivities as low as 12% have been reported for the detection of urothelial carcinoma of the ureter [50]. Ultrasound can often identify secondary signs of ureteric tumors such as hydronephrosis and hydroureter.

Many protocols for the evaluation of haematuria have used a combination of ultrasound and IVU with success for a comprehensive assessment of the upper urinary tract [51]. Recent guidelines from the European Society of Urogenital Radiology state that ultrasound may be used alone in low-risk patients to image the upper urinary tract but in high-risk patients CT urography is warranted [52].

Ultrasound is also only moderately sensitive for the detection of renal calculi (67 to 77% sensitive) [53]. Typically, renal cell carcinomas (RCC) are solid with 50% being hyperechoic to renal parenchyma and 40% being isoechoic. Urothelial carcinomas tend to be slightly hyperechoic relative to the surrounding renal parenchyma, occur more centrally, and are often associated with focal hydronephrosis [54].

## 7. Retrograde Pyelography

**Figure 4:**
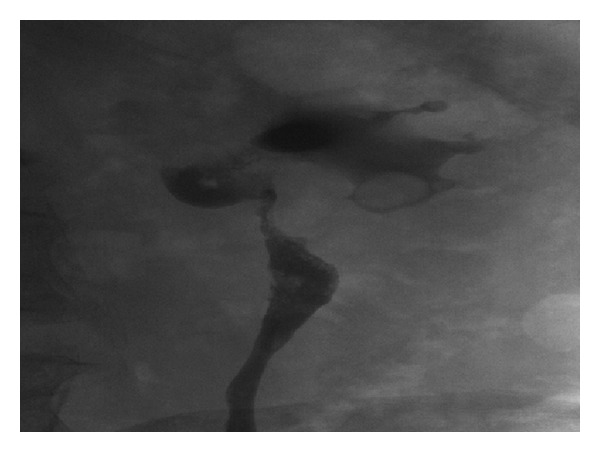
Retrograde pyelogram. There is an irregular infiltrative mass involving the renal pelvis and proximal ureter. This was a histologically proven urothelial cell carcinoma.

Retrograde pyelography can image the renal pelvis and ureters with a high degree of spatial resolution (Figure 4). However, in recent years, CTU has been found to have a greater diagnostic accuracy than retrograde pyelography for the detection of urothelial lesions [23, 55]. The high contrast resolution of CTU offers excellent pelvicaliceal and ureter visualization as well as visualization of the periureteral tissues and retroperitoneum [41]. Retrograde pyelography may still be employed as a second-line investigation to further characterize filling defects detected on other modalities, or in patients with renal failure or cases of contrast medium allergy.

## 8. Computed Tomography

The role of CT in imaging the urinary tract has expanded in recent years, particularly with the advent of multidetector (MDCT) scanners and CT urography (CTU). Contrast-enhanced CT is firmly established as the overall most sensitive modality for determining the cause of haematuria [41]. It is the gold standard in the detection of renal parenchymal masses, calculi, upper tract urothelial tumors, and extrinsic lesions [14].

**Figure 5:**
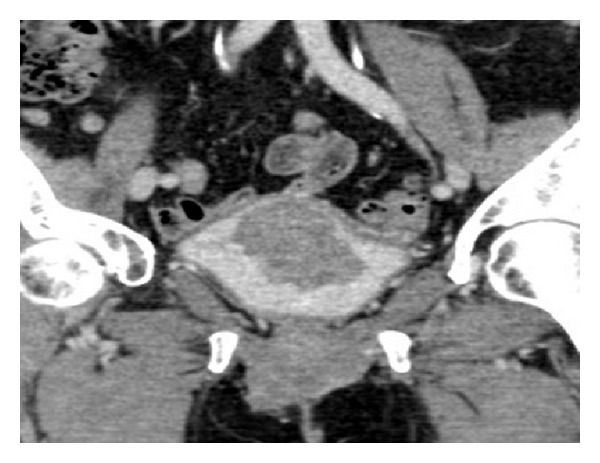
CT urogram (coronal urographic phase image) demonstrates a large polypoid mass arising from the bladder wall. This was confirmed to be a urothelial cell carcinoma following biopsy at cystoscopy.

**Figure 6:**
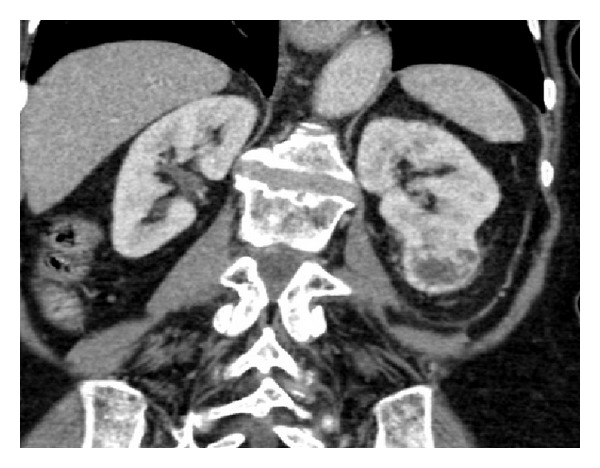
CT of kidneys (coronal nephrographic phase image) demonstrates an enhancing mass lesion arising from the lower pole of the left kidney consistent with a histologically confirmed renal cell carcinoma.

**Figure 7:**
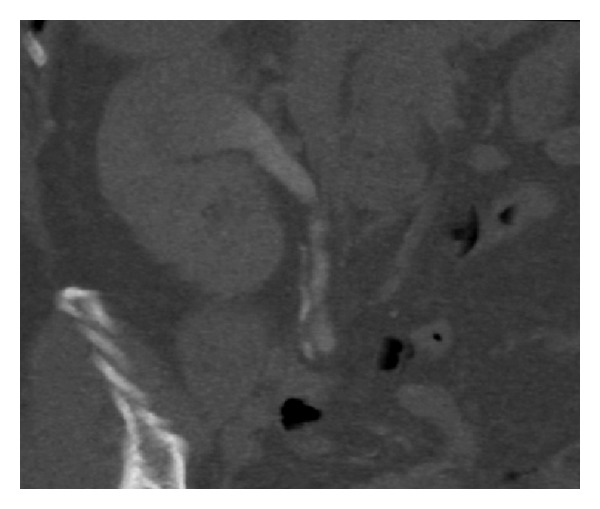
CT urogram (coronal urographic phase image) demonstrates a filling defect in the upper moiety of a duplex right kidney. This was histologically confirmed to be urothelial cell carcinoma following ureteroscopy and biopsy.

In patients presenting with acute renal colic, CT KUB will identify ureteric and bladder calculi with a sensitivity of 96% to 100% and a specificity of 94% to 100% [27, 28]. Multiphase contrast-enhanced CT is also more sensitive than IVU and ultrasound in detecting renal masses with a sensitivity of 94% compared to 67% for IVU and 79% for ultrasound [40]. It also provides excellent lesion characterization as well as imaging the adjacent retroperitoneum and providing information about the local and distant spread of malignancies (Figures 5, 6, and 7) [56]. CTU has a greater sensitivity in diagnosing upper tract urothelial malignancy than IVU, with a reported sensitivity of 95% compared to 75% with IVU [22].

When CT is chosen to image the upper urinary tract, the imaging protocol should be adapted to the patient and the diagnostic goals, such as the exclusion of urolithiasis or malignancy. CTU aims to generate multiphase thin-section images through the kidneys, ureters, and bladder that allow for the detection of the most common urological causes of hematuria, including calculi, renal masses, and urothelial tumors [57].

An unenhanced scan is initially performed from the upper poles of the kidneys to the lower edge of the symphysis pubis using 3 mm to 5 mm thick sections in the prone position. High attenuation oral contrast should be avoided, as dense contrast can make detection of ureteric calculi more difficult [58].

This is followed by nephrographic phase (90–100 seconds after contrast administration) imaging of the kidneys, and excretory phase imaging (8–15 minutes after contrast administration) of the entire urinary tract. Additional urograms including prone and oblique projection images and further delayed images may be useful in imaging obstructing ureteral lesions as well as bladder lesions [59].

The unenhanced series will detect urinary tract calculi with a high degree of diagnostic accuracy. The prone position is used to discriminate free intravesical calculi from those impacted at the vesicoureteric junction. The unenhanced series is also used to determine if a cyst is hyperdense or if any solid lesions demonstrate enhancement on subsequent phases. The nephrographic phase is the optimal phase for the detection of renal parenchymal masses [41]. Two-dimensional and three-dimensional intravenous urography-like images can be obtained by reformatting excretory phase images in the coronal or sagittal planes using volume rendering or maximum intensity projection techniques [41].

Traditionally, CTU is performed as a three-phase, single-bolus examination. However, many now advocate the use of a two-phase, split bolus technique, as it can significantly reduce the radiation dose incurred by the patient while maintaining diagnostic accuracy [60].

The use of dual energy CT urography has also been described recently with centers performing single-phase dual energy CT with synchronous nephrographic-excretory phases and reporting dose savings of up to 45% compared to standard dual-phase protocols [61].

Dose savings have also been reported with the use of material decomposition images generated from spectral CT urography allowing omission of the unenhanced scan without compromising on the detection of calculi [62].

Several authors have reported poor opacification of distal ureteric segments during CTU. Many different adjuncts have been added to CTU protocols in an attempt to improve ureteric opacification and distension. Several studies have examined the role of oral or intravenous hydration of the patient with normal saline prior to the examination, but this technique has not been widely adapted with most concurring that it does not improve opacification of the ureters [63].

Furosemide administration in low doses has been shown to be an effective and convenient technique for improving ureteric opacification. A recent comparison of intravenous furosemide and saline infusion prior to CTU found furosemide to be superior with complete ureteric opacification reported in 93% of cases [64].

Intravenous furosemide has also been used to detect and characterize renal calculi in mixed nephrographic excretory phase dual energy CT [65].

The primary deterrent limiting universal acceptance of CTU as the first-line investigation in patients with haematuria is the radiation dose which incurred. A mean effective dose of 14.8 mSv plus or minus 9 mSv has been reported for a standard three-phase CTU compared to IVU with a reported dose range of 3.6 mSv to 9.6 mSv [38].

The use of two-phase studies, split-bolus techniques, or even one-phase studies in low risk patients can all reduce radiation dose [14]. Single-phase dual-energy CT has been shown to represent an accurate “all-in-one” approach to the investigation of haematuria with a radiation dose reduction of 45% compared with a standard dual-phase protocol [61]. This technique involves the reconstruction of virtual unenhanced images from enhanced images and allows for fast and accurate characterization of renal masses in a single-phase acquisition [66].

Other disadvantages of CT include its unavailability in some centers, the risk of allergic reaction and nephrotoxicity with contrast administration, and its cost compared to IVU and ultrasound [12].

Renal cell carcinoma demonstrates early enhancement and washout following contrast material administration. Renal cell carcinoma, being hypervascular, tends to enhance more than urothelial carcinoma, although it is often difficult to differentiate the two tumors [20]. Enhancement of greater than 10 HU compared to unenhanced images suggests that a mass is solid while enhancement of greater than 20 HU strongly suggests that a mass is malignant [67].

Renal pelvis urothelial carcinoma may be seen as sessile filling defect that expands centrifugally compressing and displacing renal sinus fat or present as a pelvicaliceal irregularity, focal or diffuse mural thickening, or as an oncocalyx [54]. Ureteric lesions typically present with eccentric or circumferential wall thickening, luminal narrowing, or hydronephrosis to the level of the lesion [54].

CTU clearly outperforms conventional radiography, ultrasound, and IVU in the detection of renal parenchymal masses and urinary tract calculi. However, the relatively high radiation dose involved has prevented CTU from becoming the first-line test in all patients with haematuria.

## 9. Magnetic Resonance Urography

MRU is an evolving technology with the potential to provide a noninvasive “one-stop shop” comprehensive evaluation of the upper urinary tract and surrounding structures without the use of ionizing radiation. MR imaging offers inherently high soft-tissue contrast, is independent of excretory function, and allows multiplanar imaging, without the need for ionizing radiation.

MRI has been shown to be comparable to CT in the detection of renal masses (Figure 9) [68]. As with CT, MRI can demonstrate tumor involvement of the renal parenchyma and perinephric tissues as well as detecting distant metastases.

A significant short falling of MRI is its relative insensitivity for the detection of urinary tract calculi. In one series in which MRU was compared with noncontrast CT, MRU was inferior in the detection of calculi but detected a greater number of secondary signs of obstruction. It may be of value in situations in which exposure to ionizing radiation is undesirable [69, 70]. The sensitivity of MRU in detecting urothelial lesions also remains unclear but at present is not believed to be equivalent to IVU or CTU [59]. It also has only a moderate accuracy for the detection of bladder carcinoma [71]. Other disadvantages of MRI include its relatively long imaging times, limited availability, cost, sensitivity to motion, susceptibility to artifacts, and lower spatial resolution compared with CT and conventional radiography.

**Figure 8:**
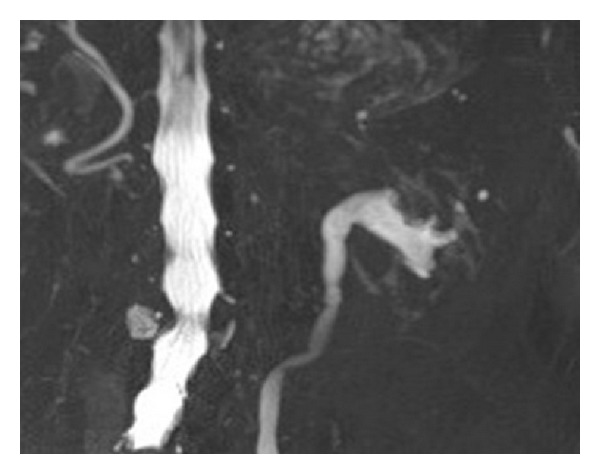
T2-weighted MR urography demonstrates a mass at the upper pole of the left kidney directly invading the renal pelvis.

**Figure 9:**
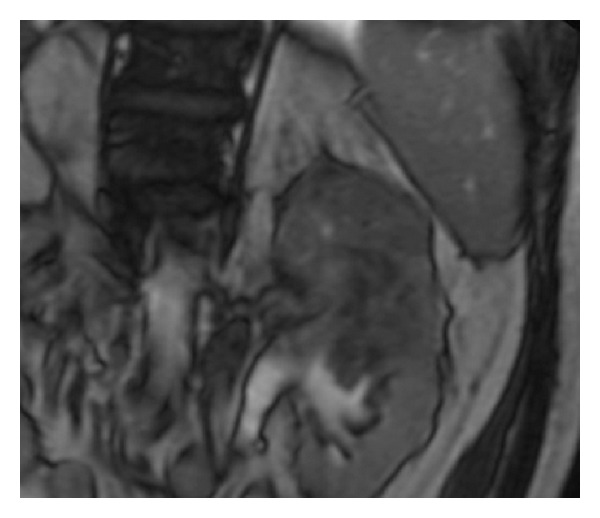
Coronal T2-weighted MRI of kidneys shows a large heterogeneous low T2 signal mass centered in the upper pole of the left kidney.

MRU has a specific role in the evaluation of painful hydronephrosis in pregnancy. Not only can it image the urinary tract without the need for ionizing radiation or contrast administration, but it can also reliably distinguish between physiological and calculus obstruction (Figure 8) [72].

MR urographic techniques used to display the urinary tract include static-fluid MRU (also known as T2-weighted MR urography) and excretory MRU (also known as T1-weighted MR urography) [70].

Static-fluid MRU techniques closely resemble those used for T2-weighted MR cholangiopancreatography and use heavily T2-weighted sequences to generate IVU-like images of the urinary tract.

Static-fluid MRU does not require the excretion of contrast material and is therefore useful for demonstrating the collecting system of an obstructed, poorly excreting kidney. Excretory MR urography is roughly analogous to IVU and CTU and uses gadolinium-enhanced T1-weighted sequences to depict the urinary tract [70].

Urinary tract calculi appear as filling defects on both static-fluid and excretory MR urography when surrounded by urine or contrast material.

At MR urography, ureteric tumors typically appear as an irregular mass which demonstrates contrast enhancement, distinguishing them from calculi [54].

Renal cell carcinoma has variable signal characteristics on T1- and T2-weighted sequences and demonstrates early enhancement following contrast administration. Renal pelvis urothelial tumors are normally isointense to renal parenchyma on T1- and T2-weighted images and are best visualized as low-signal filling defects on heavily T2-weighted or excretory contrast-enhanced T1-weighted images [73].

MRU currently serves as an alternative imaging technique to IVU and CT in radiation-sensitive patients such as pregnant women and children and in patients with contraindications to iodinated contrast media. It has not been widely adopted into clinical practice or thoroughly evaluated for effectiveness and is not currently recommended as a first-line examination.

## 10. Conclusion

Many authorities cite a lack of sufficient evidence to draw firm conclusions and formulate evidence-based guidelines on the best imaging modality to evaluate haematuria.

Conventional radiography has no role in the investigation of haematuria. Ultrasound remains an important diagnostic tool for the evaluation of haematuria in radiation-sensitive populations and low-risk patients. Retrograde pyelography remains useful for characterizing filling defects detected on other modalities, such as IVU and CTU. MRU is emerging as a potentially noninvasive comprehensive imaging test for evaluating the upper urinary tract without the use of ionizing radiation and thus is particularly useful in children and pregnant women.

It is widely accepted that CTU outperforms conventional radiography, ultrasound, and IVU in the detection of renal parenchymal masses and urinary tract calculi.

The main limitation of CTU is the associated radiation exposure and this was initially a major factor which prevented it from becoming universally accepted as the first-line investigation in patients with haematuria. Recent guidelines from the European Society of Urogenital Radiology have suggested that CTU may be used in high-risk patients in whom the risk of malignancy outweighs the risk of radiation exposure [52]. CT serves as a “one-stop shop” imaging test for patients, thereby saving time and hospital visits and leading to earlier diagnosis. Examination protocols should be tailored to the patient while all the while optimizing radiation dose.
